# Can a Surgical Vulnerability Score Predict Outcomes of Hip Reconstruction in Children with Severe Neuromuscular Disability?

**DOI:** 10.1007/s43465-024-01257-6

**Published:** 2024-09-16

**Authors:** Alistair Bevan, Stephanie Buchan, Alexander Aarvold, Simon Bennet, Darius Rad, Nick Le Prevost, Caroline Edwards

**Affiliations:** 1 Faculty of Medicine, University of Southampton, Southampton, UK; 2 Department of Paediatric Trauma and Orthopaedics, Southampton Children’s Hospital, Southampton, UK; 3 Community Paediatric Medical Service, Solent NHS Trust, Southampton, UK; 4 University Hospitals of Derby and Burton NHS Foundation Trust, Derbyshire, UK; 5 University Hospitals Plymouth NHS Trust, Plymouth, UK

**Keywords:** Vulnerability, Score, Neuromuscular, Hip reconstruction, Neuromuscular, VDRO, Dega osteotomy

## Abstract

**Background:**

Hip surgery is often necessary for children with severe neuromuscular disabilities to avoid chronic pain resulting from hip migration. This study correlated the Surgical Vulnerability Score (SVS), a novel measure of physiological reserve, with reconstructive hip surgery outcomes to improve shared surgical decision-making.

**Materials and methods:**

Sixty-eight cases performed by a single surgeon were retrospectively evaluated. Cases were graded according to physiological vulnerability using the SVS, which was then correlated with two outcomes: length of hospital stay (LOS) and severity of postoperative complications. The Gross Motor Function Classification System (GMFCS) level was used as a baseline comparison. Sub-analysis compared results for patients who underwent only a femoral varus derotation osteotomy (VDRO) (*n* = 48) with those who underwent a combined VDRO and Dega Pelvic Osteotomy (Dega PO) (*n* = 20).

**Results:**

Results showed that a higher SVS was associated with increased LOS (*p *= 0.001) and severity of postoperative complications (*p* = 0.0008). A greater GMFCS level was not associated with either LOS (*p* = 0.246) or the severity of postoperative complications (*p* = 0.282). For patients who underwent only a VDRO, an increase in SVS had no association with LOS (*p* = 0.483) or severity of complications (*p* = 0.981). However, for patients who underwent both a VDRO and Dega PO, a higher SVS was associated with increased LOS (*p* = 0.0002) and severity of complications (*p* = 0.0001).

**Conclusions:**

The SVS can aid surgical decision-making and prepare the child’s family for surgery. Early intervention and fixation using only a VDRO may lead to better outcomes, underscoring the importance of hip surveillance programs in the early identification of migrating hips.

## Introduction

Surveillance programmes are increasingly being established for the early identification of hip subluxation in children with neuromuscular disorders [1–4]. These children frequently undergo reconstructive surgery to prevent chronic pain from progressive hip migration. Orthopaedic surgery typically involves a femoral Varus Derotation Osteotomy (VDRO). In the context of concomitant acetabular dysplasia, this may be supplemented with a containment Dega Pelvic Osteotomy (Dega PO) [5]. Studies suggest that better long-term results are achieved when all existing deformities of the femur and pelvis are corrected [6, 7]. However, a combined osteotomy is associated with a higher rate of complications [8]. 

Children with neuromuscular disorders often face additional comorbidities such as seizures, chest infections, and feeding problems. This can result in frequent and lengthy hospital admissions. The range in severity of these comorbidities poses a challenge for clinicians involved in decision making, especially as there is currently limited guidance for assessing whether a patient is a suitable candidate for surgery. Although perioperative medicine is a growing speciality for adults, there are limited risk assessment techniques for children, especially those with severe disabilities [9, 10].

Hip reconstructions in cerebral palsy now have a low recorded mortality rate, but there is a significant risk of complications, and the postoperative stay can be extensive [11–13]. Children who are medically vulnerable are at the highest risk of adverse outcomes from the surgical insult of a hip reconstruction. However, inaction is difficult to justify given that a unilateral dislocation in spasticity will inevitably become painful [14, 15]. Moreover, children with severe neurodisability may have a limited life expectancy and may not benefit from long term pain relief [16].

There is existing criteria from a regional paediatric hospice to help predict mortality for the most severely affected children [17]. Termed the ‘Helen House Criteria’, this scoring system is used to grade physiological vulnerability to help identify patients who would benefit most from paediatric hospice care. As this is a measure of the physiological reserve, it may also be relevant to perioperative assessment in a similar way that perioperative exercise testing can be used for high-risk adults [18]. This criterion, termed the Surgical Vulnerability Score (SVS), has been modified to be more surgically applicable, create a numerical value and be easy to use.

This study aims to evaluate the correlation between the SVS and surgical outcomes of hip reconstruction in children with severe neurodisability. If there is any correlation, this simple assessment could help inform shared surgical decision-making for this challenging group of patients.

## Materials and Methods

### Study Population

This study was a retrospective review of patient notes on their physiological vulnerability prior to surgery, following a review of their comorbidities. 71 children with a neuromuscular disability, who underwent hip reconstruction surgery by a single surgeon at our institution between January 2014 and May 2016, were included. Three patients were excluded: one being over 18 years of age at the time of surgery, and two due to incomplete availability of their medical history. Patient demographics were recorded: Gross Motor Function Classification System (GMFCS) level, age at surgery, and surgical operation (VDRO versus combined VDRO and Dega PO).

In accordance with established protocols at our institution, pure epidural analgesia was administered to all patients for up to 72 h after surgery. A dedicated pain management team provided ongoing support, and administered oral morphine as required. Paediatric Neurologists were responsible for monitoring and managing muscle tone, with low-dose diazepam administered as deemed appropriate. Following the surgical procedure, patients were immobilised in an abduction wedge for a duration of 6 weeks.

### Surgical Vulnerability Score

The Surgical Vulnerability Score (SVS) is a numerical scoring system that originated from the ‘Helen House Criteria’, a set of evidence-based factors that affect the life expectancy of children with cerebral palsy and determine their eligibility for hospice services [17]. The SVS was further developed by adding Pain as an additional physiological category, recognizing it as a crucial marker of physiological vulnerability, particularly during the preoperative evaluation [19]. Additionally, dystonia was identified as a significant indicator of severe neurological involvement and was included as an independent neurological factor in the score [20].

**Fig. 1:**
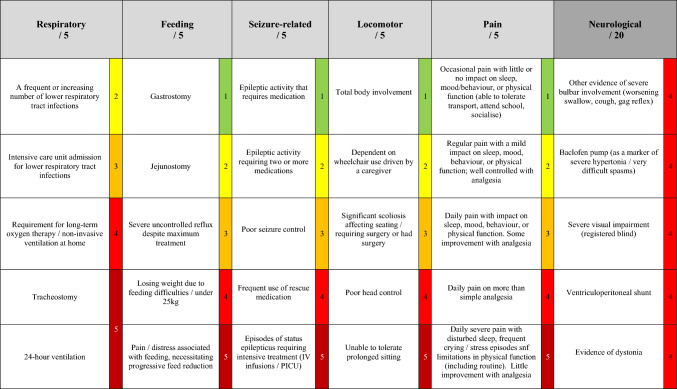
Surgical vulnerability score (SVS). This is a tool designed to assess the physiological vulnerability of patients undergoing surgery

The SVS comprises five physiological categories, each with a maximum score of 5: Respiratory, Feeding, Seizures, Locomotor, and Pain. In addition, each individual Neurological factor is awarded a score of 4, resulting in a total score out of 45 (refer to Fig. 1). A higher score indicates greater physiological vulnerability. The resulting SVS was reviewed and agreed upon by the paediatric orthopaedic team at our institution.

### Outcomes

The adverse outcomes recorded were measurable and relevant: the length of stay (LOS), postoperative complications, and mortality. LOS was recorded from the date of surgery to the date of discharge. Inpatient discharge summary and follow-up clinic notes (six weeks and three months) were reviewed for postoperative complications. Surgical survival (six weeks) and survival at the time of data collection (minimum 54 months follow-up) were also recorded. Postoperative complications were evaluated using the Clavien-Dindo classification system, whereby adverse events are graded from I to V relative to their severity (Table 1) [21].

**Table 1:** Clavien-Dindo classification system

Grade	Definition
I	Any deviation from the normal postoperative course, without the need for pharmacological treatment or surgical, endoscopic, and radiological interventions Acceptable therapeutic regimes are drugs such as antiemetics, antipyretics, analgesics, diuretics, electrolytes, and physiotherapy This grade also includes wound infections opened at the bedside
II	Requiring pharmacological treatment with drugs other than such allowed for grade I complications. Blood transfusions and total parenteral nutrition are also included
III	Requiring surgical, endoscopic, or radiological intervention
IV	Life-threatening complications (including CNS complications) requiring intensive care management
V	Death of a patient

^(19)^ This is a widely accepted classification system that classifies surgical complications according to the degree of therapy necessary to resolve them

### Statistical Analysis

IBM SPSS statistics was used to perform analysis. Regression analysis was used to correlate the SVS with both outcomes. The Pearson correlation coefficient (*r*) was calculated to describe the strength and direction of the linear association between the SVS and each variable. The determination coefficient (*R*^2^) was calculated to estimate the proportion of variability in each outcome that can be attributed to the SVS. The null hypothesis stated that there was no correlation between the SVS and each surgical outcome. A *p*-value of ≤ 0.05 was considered statistically significant. To establish a baseline for comparison, GMFCS level was correlated with outcomes to determine if the SVS was a stronger predictor of surgical results [22].

Subsequently, a subanalysis was performed for patients who underwent femoral osteotomies (VDRO) *versus* those who underwent combined femoral and pelvic osteotomies (VDRO and PO).

## Results

### Study Population

Over the 28 month study period, 68 hip reconstruction surgeries met the inclusion criteria, of which 70.6% (*n* = 48) were femoral VDROs and 29.4% (*n* = 20) were combined VDRO and Dega PO surgeries. The distribution of GMFCS for the cohort was Level II–5 (7.4%), Level III–5 (7.4%), Level IV–25 (36.8%), Level V–33 (48.5%). The mean age at surgery was 11.9 years (SD = 3.7), ranging from 3.0 to 18.0 years, and 29.4% of patients had documented evidence of dystonia. Of the 68 cases, 65 patients (95.6%) had Cerebral Palsy, 2 (2.9%) had Myelomeningocele and 1 (1.5%) had Muscular Dystrophy.

### Surgical Outcomes

**Fig. 2:**
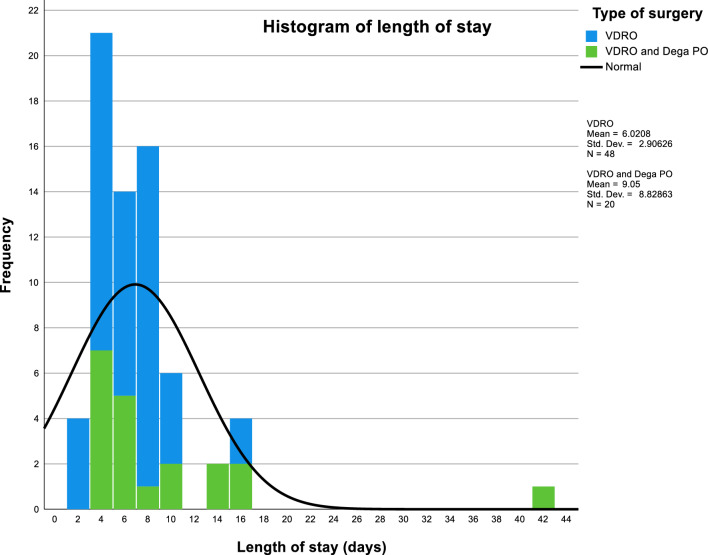
Histogram showing the postoperative length of stay, subdivided by the type of hip surgery the patient underwent. The normal distribution is also shown

The median SVS of the cohort was 7.0, with a range of 0–21 and an interquartile range (IQR) of 4–10 (skewness = 0.7). The median length of stay was 6.0 days, ranging from 2 to 42 days, with an IQR of 4–8 days (skewness = 4.2) (Fig. 2). Out of the 68 surgeries, 17 cases (25.0%) experienced complications of varying severity, with a range of Clavien-Dindo grade 1–4, and a mean severity of 2.1 (SD = 1.1) (Table 2). There were no mortalities at the final follow-up, which was a minimum of 54 months.

**Table 2:** Table detailing the seventeen recorded complications

Surgery	Adverse outcome	Clavien-Dindo grade
Isolated VDRO	Mild pressure sores	1
	Moderate pressure sores	
	Respiratory distress	
	Postoperative hip pain	
	Postoperative hip pain	
	Postoperative pain in the right flank	
	Blocked Gastrostomy-jejunostomy tube, pneumonia	2
	Postoperative anaemia (requiring blood transfusion)	
	Postoperative pain (received Botox injections)	3
	Failed hip fixation (corrected surgically)	
	Community-acquired pneumonia (urgent removal of the spica cast)	
VDRO + Dega PO	Open wound incision	1
	Aspiration pneumonia	2
	Ileus (treated in theatre)	3
	Excessive external rotation of the right hip, flexion abduction pattern in the left hip (corrected surgically)	
	The collapse of the left upper and lower lung lobe fields (PICU admission)	4
	Seizures, infected tracheostomy site, and food intolerance (PICU admission)	

Each complication was classified according to severity using the Clavien-Dindo classification system

### Correlations

Regression analysis showed a significant positive correlation between SVS and both the LOS (*r* = 0.383, *R*^2^ = 0.147, *p*-value = 0.001) and the severity of postoperative complications (*r* = 0.396, *R*^2^ = 0.157, *p*-value = 0.0008). There was only a mild positive correlation between GMFCS level and both the LOS (*r* = 0.143, *R*^2^ = 0.020, *p*-value = 0.246) and the severity of postoperative complications (*r* = 0.132, *R*^2^ = 0.018, *p*-value = 0.282), which were not statistically significant.

### Sub-Analysis by Operation

Of the 48 isolated VDROs, 25 (54.0%) were performed unilaterally, while 24 (46.0%) were bilateral. Among the 20 combined VDRO with Dega PO, 10 (50.0%) were performed unilaterally, 3 (15.0%) were bilateral VDROs with unilateral Dega PO, and 7 (35.0%) were bilateral VDROs with bilateral Dega PO. The age at surgery and sex ratio were comparable across surgical subgroups (independent T test; conditions: *t*(45.1) = − 1.29, *p* = 0.203) with male–female ratios of 58.3%-41.7% for VDRO, and 60.0%-40.0% for VDRO and Dega PO.

The distribution of GMFCS for patients who underwent a VDRO and Dega PO was Level IV–7 (35.0%), Level V–13 (65.0%). The GMFCS of patients who underwent an isolated VDRO was Level II–5 (10.4%), Level III–5 (10.4%), Level IV–18 (37.5%), Level V–20 (41.7%). Patients who received a VDRO and Dega PO had a higher SVS; mean = 9.6 (SD = 5.3), minimum = 2, maximum = 21, IQR = 6.5–11.3 (skewness = 0.8) compared to those who received only a VDRO; mean = 6.5 (SD = 4.6), minimum = 2, maximum = 15, IQR = 3–9 (skewness = 0.6). Evidence of dystonia was observed in 9 (18.8%) of the patients who underwent an isolated VDRO compared to 11 (55.0%) patients in the group that underwent a VDRO and Dega PO.

**Fig. 3:**
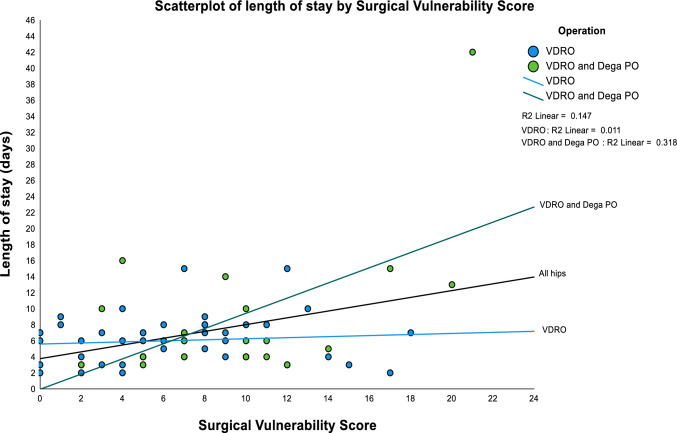
Scatter plot of length of stay *versus* the Surgical Vulnerability Score. This plot also includes subcategorization by the method of reconstructive hip surgery. The coefficient of determination (*R*^2^) has been plotted for all cases (*n* = 68), and for each subcategory

Among the 48 isolated VDROs, 11 (22.9%) recorded complications with a mean severity grade of 1.7 (SD = 0.9). The median length of stay (LOS) for this group was 6.0 days (SD = 2.9), with a range of 2–15 days and an IQR of 4–7.3 days (skewness = 1.1). Although regression analysis showed a mild positive correlation between SVS and both LOS (*r* = 0.104, *R*^2^ = 0.011, *p*-value = 0.483) (Fig. 3) and severity of postoperative complications (*r* = 0.055, *R*^2^ = 0.003 *p*-value = 0.981), these correlations were not statistically significant.

Among the 20 combined VDRO and Dega PO operations, 6 (30.0%) reported complications with a mean severity grade of 2.8 (SD = 1.2). The median length of stay (LOS) for this group was 6.0 days, but it was skewed by a single case that recorded an inpatient stay of 42 days. The smallest LOS was 3 days, with an IQR range of 4–12.3 days and a skewness of 3.0. Regression analysis revealed a statistically significant positive correlation between SVS and both the LOS (*r* = 0.564, *R*^2^ = 0.318, *p*-value = 0.0002) (Fig. 3) and the severity of postoperative complications (*r* = 0.760, *R*^2^ = 0.577, *p*-value = 0.0001).

## Discussion

The effectiveness of numerous surveillance programs worldwide in preventing the migration of neuromuscular hips and the consequent development of symptoms has been well-documented [23–25]. The SARS-CoV-2 pandemic has highlighted the devastating impact of inadequate surveillance programs on the paediatric neuromuscular population, as evidenced by cases of hips that have migrated beyond the point of salvaging [26]. The exact prevalence and extent of this phenomenon remain uncertain, underscoring the need for further research in this area.

Reconstructive hip surgery has been proven to significantly improve the quality of life for children with neuromuscular disability [27–30]. This study reports no mortality within the follow-up period, supporting evidence that most children will live long enough to experience benefits from their operation [11–13]. However, this study highlights the considerable burden of comorbidities associated with these patients and the risk involved in major surgery. Thus, a reliable assessment tool that can identify individuals at higher risk for adverse outcomes is urgently needed. Such a tool would enable the surgical team to proactively plan for complex cases by arranging multidisciplinary meetings and securing post-operative facilities that are optimally equipped to manage these patients, including high dependency or intensive care units.

The SVS can provide a framework to quantify this burden. This study found that a higher score was correlated with an increased length of stay and severity of postoperative complications. Thus, the SVS could be used to identify children at increased risk of adverse outcomes, allowing for more informed surgical consent and better preparation for the surgical journey. It could also serve as a teaching tool to assist healthcare professionals in identifying and monitoring areas of physiological weakness and tracking comorbid progression.

In comparison, although simple to assess, GMFCS level fails to account for the majority of severe comorbidities these children may have. This study found an increase in GMFCS level had no correlation with the length of stay and severity of postoperative complications, making it a less relevant predictor of outcomes than the SVS in perioperative medicine. Further, *Wagner and Hägglund* (2022) found a non-significant correlation (*p*-value = 0.9) between the GMFCS level and the development of hip migration [31].

Subanalysis showed patients who underwent a combined VDRO and Dega PO had a higher mean SVS compared to those who underwent an isolated VDRO. A combined osteotomy also had a comparatively high complication rate and mean length of stay. This is unsurprising considering the greater surgical insult caused by the addition of a pelvic osteotomy [8].

For the combined VDRO and Dega PO, a higher SVS was strongly correlated with adverse surgical outcomes. On the contrary, for patients who underwent an isolated VDRO, a higher SVS had no correlation with adverse outcomes. This finding suggests that an isolated VDRO may be a more tolerable operation for patients with significant comorbidities. Perhaps the greater surgical insult of the combined femoral and pelvic osteotomy is enough to adversely affect those with less physiological reserve.

However, performing an isolated VDRO may not always be feasible, as it is not sufficient to achieve and maintain a reduction in the context of acetabular dysplasia [7, 32]. Therefore, the results of this study suggest that an earlier intervention, when only a VDRO is indicated, would result in fewer complications. This finding further demonstrates the value of hip surveillance programmes for children with neuromuscular disability. Surveillance programmes can identify progressively migrating hips, allowing early surgical intervention [1–4]. For children who do go on to require a combined osteotomy, the SVS can identify those at increased risk of adverse outcomes to enable a closer postoperative follow-up.

This study is limited by its retrospective design and its scope of evaluation of adverse outcomes, being intentionally limited to length of stay, complications, and mortality. The cohort size is comparable to many studies on surgery for children with neuromuscular disability but is still small. Subanalysis by operation resulted in yet smaller group sizes. We were unable to perform Subanalysis by laterality due to the small cohort size. This study did not factor in surgical time, type of anaesthesia used, blood loss and post-operative mobilisation.

Furthermore, as the GMFCS was specifically designed for children with cerebral palsy, its applicability to other conditions is limited. While it has been used to assess gross motor function in both muscular dystrophy [33–35] and myelomeningocele [34–38], it lacks validation in these populations. Consequently, its use as a direct comparator in this study is constrained.

The SVS, which is an entirely novel tool, has not yet been evaluated. Thus, a power calculation was not possible. It was unable to detect adverse outcomes in patients who underwent an isolated VDRO, which is still a major surgery. Although statistically significant correlations were identified between SVS and outcomes for the group that underwent combined surgery, the *R*^2^ values suggested a weak or moderate relationship. Therefore, more work is needed to validate this scoring system and make it widely applicable. Further research should aim to determine a critical value for the SVS, at which point an early VDRO may be indicated.

In summary, the SVS has the potential to aid surgical decision-making for healthcare professionals and families, allowing for more informed consent and better preparation for the surgical journey.

## Data Availability

The data that support the findings of this study are available from the corresponding author, AB, upon reasonable request.

## References

[1] Juan, A. M. S., & Swaroop, V. T. (2022). Cerebral palsy: hip surveillance. *Pediatric Annals,**51*(9), e353–e356. 36098607 10.3928/19382359-20220706-06

[2] Gaston, M. S., Wordie, S. J., Wagner, P., Hägglund, G., & Robb, J. E. (2022). The CPUP hip score predicts displacement of the hip in children with cerebral palsy. *The Bone Joint Journal,**104-B*(5), 640–4. 35491586 10.1302/0301-620X.104B5.BJJ-2021-0301.R5

[3] Wordie, S. J., Robb, J. E., Hägglund, G., Bugler, K. E., & Gaston, M. S. (2020). Hip displacement and dislocation in a total population of children with cerebral palsy in scotland. *Bone Joint Journal,**102-b*(3), 383–7. 32114804 10.1302/0301-620X.102B3.BJJ-2019-1203.R1

[4] Miller, S. D., Shore, B. J., & Mulpuri, K. (2019). Hip surveillance is important to children with cerebral palsy: stop waiting, start now. *Journal of American Academy Orthopaedic Surgeons Global Research and Reviews,**3*(4), e021. 10.5435/JAAOSGlobal-D-19-00021 PMC6510464 31334474

[5] Reidy, K., Heidt, C., Dierauer, S., & Huber, H. (2016). A balanced approach for stable hips in children with cerebral palsy: a combination of moderate VDRO and pelvic osteotomy. *Journal of Children’s Orthopaedics,**10*(4), 281–288. 27349432 10.1007/s11832-016-0753-5 PMC4940248

[6] Al-Ghadir, M., Masquijo, J. J., Guerra, L. A., & Willis, B. (2009). Combined femoral and pelvic osteotomies versus femoral osteotomy alone in the treatment of hip dysplasia in children with cerebral palsy. *Journal of Pediatric Orthopedics,**29*(7), 779–783. 20104162 10.1097/BPO.0b013e3181b76968

[7] Miller, F. (2017). Hip Reconstruction in Children with Cerebral Palsy. In F. Miller, S. Bachrach, N. Lennon, & M. O’Neil (Eds.), *Cerebral Palsy* (pp. 1–27). Springer International Publishing.

[8] Czubak, J., Kowalik, K., Kawalec, A., & Kwiatkowska, M. (2018). Dega pelvic osteotomy: indications, results and complications. *Journal of Children’s Orthopaedics,**12*(4), 342–348. 30154924 10.1302/1863-2548.12.180091 PMC6090189

[9] Grocott, M. P. W., Edwards, M., Mythen, M. G., & Aronson, S. (2019). Peri-operative care pathways: re-engineering care to achieve the ‘triple aim.’ *Anaesthesia,**74*(Suppl 1), 90–99. 30604413 10.1111/anae.14513

[10] Levett, D. Z., Edwards, M., Grocott, M., & Mythen, M. (2016). Preparing the patient for surgery to improve outcomes. *Best Practice and Research. Clinical Anaesthesiology,**30*(2), 145–157. 27396803 10.1016/j.bpa.2016.04.002

[11] Ruzbarsky, J. J., Beck, N. A., Baldwin, K. D., Sankar, W. N., Flynn, J. M., & Spiegel, D. A. (2013). Risk factors and complications in hip reconstruction for nonambulatory patients with cerebral palsy. *Journal of Children’s Orthopaedics,**7*(6), 487–500. 24432112 10.1007/s11832-013-0536-1 PMC3886352

[12] DiFazio, R., Vessey, J. A., Miller, P., Van Nostrand, K., & Snyder, B. (2016). Postoperative complications after hip surgery in patients with cerebral palsy: a retrospective matched cohort study. *Journal of Pediatric Orthopedics,**36*(1), 56–62. 25633609 10.1097/BPO.0000000000000404

[13] Spencer, J. D. (1999). Reconstruction of dislocated hips in children with cerebral palsy. *BMJ,**318*(7190), 1021–1022. 10205082 10.1136/bmj.318.7190.1021 PMC1115434

[14] Hägglund, G., Alriksson-Schmidt, A., Lauge-Pedersen, H., Rodby-Bousquet, E., Wagner, P., & Westbom, L. (2014). Prevention of dislocation of the hip in children with cerebral palsy: 20 year results of a population-based prevention programme. *Bone Joint Journal,**96-B*(11), 1546–52. 25371472 10.1302/0301-620X.96B11.34385

[15] Hägglund, G., Lauge-Pedersen, H., & Wagner, P. (2007). Characteristics of children with hip displacement in cerebral palsy. *BMC Musculoskeletal Disorders,**8*, 101. 17963501 10.1186/1471-2474-8-101 PMC2194677

[16] Abuga, J. A., Kariuki, S. M., Kinyanjui, S. M., Boele van Hensbroek, M., & Newton, C. R. (2021). Premature mortality, risk factors, and causes of death following childhood-onset neurological impairments: a systematic review. *Frontiers Neurology,**12*, 627824. 10.3389/fneur.2021.627824 PMC8062883 33897590

[17] Harrop E, Brombley K (2012) Vulnerability Factors considered for acceptance of children with Cerebral Palsy or other static neurological conditions to children’s hospice services Togetherforshortlives.org.uk 2012 [Available from: https://www.togetherforshortlives.org.uk/wp-content/uploads/2018/01/ExRes-Vulnerability-factros-for-acceptance-of-children-with-cerebral-palsy.pdf.

[18] Levett, D. Z., & Grocott, M. P. (2015). Cardiopulmonary exercise testing for risk prediction in major abdominal surgery. *Anesthesiology Clinics,**33*(1), 1–16. 25701925 10.1016/j.anclin.2014.11.001

[19] Erlenwein, J., Przemeck, M., Degenhart, A., Budde, S., Falla, D., Quintel, M., et al. (2016). The influence of chronic pain on postoperative pain and function after hip surgery: a prospective observational cohort study. *The Journal of Pain,**17*(2), 236–247. 26548971 10.1016/j.jpain.2015.10.013

[20] Supiot, F. (2017). Identification and measurement of dystonia in cerebral palsy. *Developmental Medicine and Child Neurology,**59*(12), 1211. 28892136 10.1111/dmcn.13543

[21] Dindo, D., Demartines, N., & Clavien, P. A. (2004). Classification of surgical complications: a new proposal with evaluation in a cohort of 6336 patients and results of a survey. *Annals of Surgery,**240*(2), 205–213. 15273542 10.1097/01.sla.0000133083.54934.ae PMC1360123

[22] Palisano, R., Rosenbaum, P., Walter, S., Russell, D., Wood, E., & Galuppi, B. (1997). Development and reliability of a system to classify gross motor function in children with cerebral palsy. *Developmental Medicine and Child Neurology,**39*(4), 214–223. 9183258 10.1111/j.1469-8749.1997.tb07414.x

[23] Shrader, M. W., Wimberly, L., & Thompson, R. (2019). Hip surveillance in children with cerebral palsy. *Journal of American Academy of Orthopaedic Surgeons,**27*(20), 760–768. 10.5435/JAAOS-D-18-00184 30998565

[24] Wynter, M., Gibson, N., Willoughby, K. L., Love, S., Kentish, M., Thomason, P., et al. (2015). Australian hip surveillance guidelines for children with cerebral palsy: 5 year review. *Developmental Medicine and Child Neurology,**57*(9), 808–820. 25846730 10.1111/dmcn.12754

[25] Hägglund, G., Andersson, S., Düppe, H., Lauge-Pedersen, H., Nordmark, E., & Westbom, L. (2005). Prevention of dislocation of the hip in children with cerebral palsy: the first ten years of a population-based prevention programme. *The Journal of Bone and Joint Surgery British,**87*(1), 95–101. 15686244

[26] Wordie, S. J., Bugler, K. E., Bessell, P. R., Robb, J. E., & Gaston, M. S. (2021). Hip displacement in children with cerebral palsy. *The Bone & Joint Journal,**103-B*(2), 411–4. 33517734 10.1302/0301-620X.103B2.BJJ-2020-1528.R1

[27] DiFazio, R. L., Vessey, J. A., Miller, P. E., Snyder, B. D., & Shore, B. J. (2022). Health-related quality of life and caregiver burden after hip reconstruction and spinal fusion in children with spastic cerebral palsy. *Developmental Medicine & Child Neurology.,**64*(1), 80–87. 34296760 10.1111/dmcn.14994

[28] Sankar, W. N., Spiegel, D. A., Gregg, J. R., & Sennett, B. J. (2006). Long-term follow-up after one-stage reconstruction of dislocated hips in patients with cerebral palsy. *Journal of Pediatric Orthopedics,**26*(1), 1–7. 16439892 10.1097/01.bpo.0000190842.77036.d0

[29] Rutz, E., Vavken, P., Camathias, C., Haase, C., Jünemann, S., & Brunner, R. (2015). Long-term results and outcome predictors in one-stage hip reconstruction in children with cerebral palsy. *Journal of Bone and Joint Surgery. American Volume,**97*(6), 500–506. 25788307 10.2106/JBJS.N.00676

[30] Braatz, F., Eidemüller, A., Klotz, M. C., Beckmann, N. A., Wolf, S. I., & Dreher, T. (2014). Hip reconstruction surgery is successful in restoring joint congruity in patients with cerebral palsy: long-term outcome. *International Orthopaedics,**38*(11), 2237–2243. 24968787 10.1007/s00264-014-2379-x

[31] Wagner, P., & Hägglund, G. (2022). Development of hip displacement in cerebral palsy: a longitudinal register study of 1045 children. *Acta Orthopaedica,**93*, 124–131. 34984476 10.2340/17453674.2021.851 PMC8815423

[32] Chang, F. M., May, A., Faulk, L. W., Flynn, K., Miller, N. H., Rhodes, J. T., et al. (2018). Outcomes of isolated varus derotational osteotomy in children with cerebral palsy hip dysplasia and predictors of resubluxation. *Journal of Pediatric Orthopedics,**38*(5), 274–278. 27280898 10.1097/BPO.0000000000000809

[33] Bayram, E., Topcu, Y., Karakaya, P., Bayram, M. T., Sahin, E., Gunduz, N., et al. (2013). Correlation between motor performance scales, body composition, and anthropometry in patients with duchenne muscular dystrophy. *Acta Neurologica Belgica,**113*(2), 133–137. 22975832 10.1007/s13760-012-0125-y

[34] Kilpinen-Loisa, P., Paasio, T., Soiva, M., Ritanen, U. M., Lautala, P., Palmu, P., et al. (2010). Low bone mass in patients with motor disability: prevalence and risk factors in 59 finnish children. *Developmental Medicine and Child Neurology,**52*(3), 276–282. 19709135 10.1111/j.1469-8749.2009.03464.x

[35] Matsumoto, H., Clayton-Krasinski, D. A., Klinge, S. A., Gomez, J. A., Booker, W. A., Hyman, J. E., et al. (2011). Development and initial validation of the assessment of caregiver experience with neuromuscular disease. *Journal of Pediatric Orthopedics,**31*(3), 284–292. 21415688 10.1097/BPO.0b013e31820fc522

[36] Baker, R., McGinley, J. L., Schwartz, M. H., Beynon, S., Rozumalski, A., Graham, H. K., et al. (2009). The gait profile score and movement analysis profile. *Gait and Posture,**30*(3), 265–269. 19632117 10.1016/j.gaitpost.2009.05.020

[37] Piškur, B., Beurskens, A. J., Jongmans, M. J., Ketelaar, M., & Smeets, R. J. (2015). What do parents need to enhance participation of their school-aged child with a physical disability? a cross-sectional study in the Netherlands. *Child: Care, Health and Development,**41*(1), 84–92. 24797584 10.1111/cch.12145

[38] Williams, E. N., Carroll, S. G., Reddihough, D. S., Phillips, B. A., & Galea, M. P. (2005). Investigation of the timed ‘up & go’ test in children. *Developmental Medicine and Child Neurology,**47*(8), 518–524. 16108451 10.1017/s0012162205001027

